# Reprocessing of Contaminated MGIT 960 Cultures to Improve Availability of Valid Results for Mycobacteria

**DOI:** 10.1155/2020/1721020

**Published:** 2020-07-18

**Authors:** Balaji Subramanyam, Gomathi Sivaramakrishnan, Devi Sangamithrai, Rajkumar Ravi, Kannan Thiruvengadam, Vaishnavi Vijayaragavan, Ayswarya Selvaraju, Srikanth Prasad Tripathy, Rajesh Mondal

**Affiliations:** Department of Bacteriology, ICMR-National Institute for Research in Tuberculosis, Chennai, India

## Abstract

Optimal recovery of mycobacteria from the contaminated liquid culture is a challenge. While alternative methods have been suggested to reduce the rate of contamination in the BACTEC MGIT 960 system, reprocessing the contaminated liquid culture improves recovery of *Mycobacterium tuberculosis*. Among 793 MGIT cultures raised from as many sputum specimens after primary decontamination by the standard NaLC-NaOH method, valid results were available for 687 (86.6%) as 106 (13.4%) were contaminated. Reprocessing and reculturing of the contaminated cultures increased valid results to 739 (93.2%) and reduced the contamination rate to 6.8%. Both values were statistically significant. Recovery of the *Mycobacterium tuberculosis* complex increased from 45.6% to 48.4%. Valid negative results were available for an additional 3.4%. The method may be adopted to reduce the rate of contamination and to improve the valid culture results for mycobacteria.

## 1. Introduction

The Center for Disease Control and Prevention (CDC) recommends the use of most efficient and rapid methods available for diagnosis of tuberculosis [1]. They include the use of at least one liquid medium such as the BACTEC MGIT 960 method for the primary isolation of mycobacteria [2]. Controlling the overgrowth of normal flora present in sputum is a major challenge while culturing mycobacteria in the liquid medium. Overgrowth of normal flora leads to contamination which affects the sensitivity of detection of the method especially in temperate settings. BACTEC MGIT 960 is currently considered as the gold standard by the World Health Organization (WHO) and is used widely for primary isolation as well as drug susceptibility testing (DST) of *Mycobacterium tuberculosis* (MTB). However, high rates of contamination in MGIT 960 [3] are known to result in lower recovery rate of mycobacteria [4].

Alternatives such as use of a higher concentration of NaOH up to 1.5% for decontamination of sputum and increasing the volume of the antimicrobial supplement PANTA have been suggested by the manufacturer for reducing the rate of contamination. Reprocessing of the contaminated culture and incubating the same are also recommended but rarely practiced [5]. Failure to recover MTB hampers DST and raises concern especially if the culture is crucial for patient management and also in the case of extrapulmonary specimens.

The present work aims to report on reprocessing of the contaminated liquid culture for optimal recovery of mycobacteria and to reduce the rate of contamination from clinical specimens of a temperate endemic setting.

## 2. Materials and Methods

The observational study was done at the National Institute for Research in Tuberculosis (NIRT), Chennai, India, and was based on retrospective analysis of liquid cultures of all diagnostic and follow-up sputum specimens collected from patients under the NIRT Institutional Ethics Committee-approved studies and from patients attending clinics of the Revised National Tuberculosis Control Program requiring culture, for a period of one month (February, 2019).

A total of 793 consecutive consequent specimens comprising smear-positive and smear-negative specimens of both diagnostic and follow-up specimens were included in the study. The specimens were processed by the standard NaLC-NaOH method for digestion, decontamination, and concentration. A final concentration of 1.5% NaOH was used for processing sputum specimens. The final pellet was resuspended in 1 ml of 0.067 M phosphate-buffered saline (PBS) at pH 6.8. Primary culture on BACTEC MGIT 960 was made using 0.5 ml of the pellet as per the manufacturer's protocol. Tubes flagged positive by the MGIT 960 instrument were subjected to further identification tests for differentiation of the *Mycobacterium tuberculosis* complex from other nontuberculous mycobacteria (NTM) as per the standard protocol. In brief, cultures were subjected to AFB staining to demonstrate the presence or absence of AFB, inoculation on brain heart infusion (BHI) agar to check for growth of contaminating organisms, and the commercial MPT64 immunochromatographic test (TBc ID, Becton and Dickinson, USA) to confirm the presence of the MTB complex.

Tubes showing growth on BHI agar were subjected to reprocessing as per protocol mentioned in the manufacturer's insert [5]. In brief, the culture was treated with an equal amount of 4% NaOH for 15 minutes. Following centrifugation at 3000 ×g for 15 minutes, the pellets were neutralized using 0.067 M PBS at pH 6.8. A fresh MGIT tube was inoculated with 0.5 ml of the pellet and antibiotic supplement for the primary culture, and the tube was loaded into the MGIT instrument and followed up as for any primary culture. Any tube flagging positive was again subjected to the identification tests and results finalized. The tubes were coded before reprocessing, and the results were compared with the original primary results after decoding.

## 3. Results

### 3.1. Primary Culture

Among 793 sputum specimens processed, 362 (45.6%) were positive for the MTB complex, 296 (37.3%) specimens were negative, 29 (3.7%) were identified as nontuberculous mycobacteria (NTM), and 106 specimens were contaminated (13.4%). The availability of valid results was 687 out of 793 (86.6%) (Table 1).

### 3.2. Reprocessed Cultures

Among 106 specimens that were reprocessed and recultured, valid results were available for an additional 52 cultures (6.6%) with 22 yielding the MTB complex, 27 yielding negative results, and 3 yielding NTM growth and 54 remaining contaminated. Reprocessing of contaminated cultures resulted in statistically significant increase in the valid results from 86.6% (*n* = 687) to 93.2% (*n* = 739) and decrease in the contamination rate from 13.4% (*n* = 106) to 6.8% (*n* = 54) (*p* < 0.001) (Table 1).

## 4. Discussion

Contamination in the liquid culture by bacteria or fungi reflects the presence of microorganisms that survive the action of the decontaminating agent (NaOH) in the specimens. The most common organisms causing contamination are from respiratory sources viz. *Bacillus sp.*, *Staphylococcus sp.*, *Micrococcus sp.*, *Pseudomonas sp.*, coagulase-negative *Staphylococci*, and *Corynebacterium sp.* [6]. Presence of yeast and molds was also reported [7].

Overgrowth of normal flora in processed sputum specimens especially in liquid culture is a challenge as it affects availability of valid results that could be crucial for patient management as well as the utility of the diagnostic test. National TB programs in many countries are still dependent on the liquid culture and DST using BACTEC MGIT 960 for management of all forms of TB. Failure to isolate MTB or demonstrate the absence of MTB in sputum during crucial month cultures and in extrapulmonary TB leads to unnecessary delays in taking decisions. Furthermore, a higher contamination rate reflects poor performance by the lab.

Several efforts were made by different laboratories to minimize the rate of contamination in the liquid culture and to increase the rate of recovery of mycobacteria. Mitchison et al. in 1983 [8] used selective Kirchner's liquid medium supplemented with antibiotics along with solid Lowenstein–Jensen slopes to increase the yield of tubercle bacilli and to decrease the rate of contamination. In another attempt, vancomycin was included along with the commercial PANTA antimicrobial supplement to suppress the growth of nonmycobacterial contaminants [9]. In 2011, Peres et al. [10] assessed the effect of a double concentration of supplemental PANTA and suggested that it may be useful in reducing contamination without reducing the diagnostic yield.

Whyte et al. [11] reported that the performance of the MGIT 960 system in terms of isolation of mycobacteria is not superior to the BACTEC 460 system and that the high contamination of MGIT 960 was a major concern. The reason for higher contamination rate is attributed to the presence of rich protein in the MGIT 960 medium [12], the percentage of NaOH used to decontaminate sputum specimens [13], and climate condition and delay in transport of specimens to the laboratory [14]. However, factors such as lower time to detection, greater sensitivity than conventional LJ media, comparable sensitivity to the radiometric BACTEC 460 system in detecting mycobacteria from clinical specimens, and good concordance with both LJ and BACTEC 460 DST favor the use of the BACTEC MGIT 960 system [15] which is recommended as the gold standard.

Reprocessing of the contaminated culture and reculturing have been recommended by the manufacture of the BACTEC MGIT 960 system to improve availability of valid results and increase recovery of MTB. However, this is rarely practiced. Many laboratories resort to reinoculation of the stored pellet when the primary culture gets contaminated. This could affect valid results in two ways—loss of viability of MTB due to storage of pellet at an inappropriate temperature and growth of contaminating organisms present in the pellet as it is already demonstrated by the contaminated primary culture.

Documentation of higher valid results and MTB retrieval through practice of reprocessing and reculturing of contaminated MGIT cultures is needed to guide laboratories to implement the protocol stringently. This is one of the first such reports and demonstrates the utility of the method for application in TB laboratories.

## Figures and Tables

**Table 1:** Effect of reprocessing of the contaminated mycobacterial liquid culture.

	Before reprocessing	After reprocessing	% difference	*p* value
Contamination	106 (13.4%)	54 (6.8%)	6.60	**<0.001**
Positive for the MTB complex	362 (45.6%)	384 (48.4%)	2.80	0.286
Nontuberculous mycobacteria (NTM)	29 (3.7%)	32 (4.0%)	0.30	0.857
Negatives	296 (37.3%)	323 (40.7%)	3.40	0.181
Total	793			
Valid results^@^	687 (86.6%)	739 (93.2%)	6.60	<0.001

^@^Cumulative number of cultures yielding MTB complex, NTM, and negatives.

## Data Availability

The data (Table 1) used to support the findings of this study are included within the article.
